# Upadacitinib in the treatment of a patient with the triad of atopic dermatitis, vitiligo, and alopecia areata: a case report and literature review

**DOI:** 10.3389/fimmu.2026.1795986

**Published:** 2026-06-08

**Authors:** Tao Wang, Xiaofang Zhang, Jingping Wang, Kaiwen Zhuang, Tingting Wang

**Affiliations:** 1 Department of Dermatology, West China Hospital, Sichuan University, Chengdu, China; 2 Department of Dermatology, Mianyang 404 Hospital, Mianyang, China; 3 Laboratory of Dermatology, Clinical Institute of Inflammation and Immunology, Frontiers Science Center for Disease-related Molecular Network, West China Hospital, Sichuan University, Chengdu, China

**Keywords:** alopecia areata, atopic dermatitis, JAK-STAT pathway, upadacitinib, vitiligo

## Abstract

**Background:**

The simultaneous occurrence of severe atopic dermatitis (AD), vitiligo, and alopecia areata (AA) in a single patient is exceptionally rare and poses a significant therapeutic challenge, as conventional “disease-specific” therapies often yield suboptimal results. Emerging evidence implicates the shared JAK-STAT signaling pathway in the pathogenesis of these conditions, suggesting the potential for a targeted “multi-disease therapy” approach.

**Case presentation:**

A 57-year-old man presented with severe, treatment-refractory AD, extensive vitiligo, and AA. Following the failure of conventional therapies, treatment with the selective JAK1 inhibitor upadacitinib (15 mg daily) was initiated. A rapid and marked improvement was observed: pruritus subsided within days, AD-related erythema cleared substantially by 15 weeks, complete eyebrow regrowth occurred within months, and significant repigmentation of vitiligo lesions was evident at one-year follow-up. The treatment was well-tolerated.

**Conclusion:**

This case suggests that upadacitinib monotherapy may be an effective and well-tolerated option for severe co-existing AD, vitiligo, and AA. It provides clinical evidence supporting the novel “multi-disease therapy” paradigm, which targets common pathogenic pathways rather than individual disease phenotypes, offering a new strategic direction for managing complex immune-mediated comorbidities.

## Introduction

Atopic dermatitis (AD), vitiligo, and alopecia areata (AA) are distinct immune-mediated skin diseases that share dysregulation of the JAK-STAT signaling pathway. AD is largely driven by Th2 cytokines (e.g., IL-4, IL-13) signaling through JAK1, while vitiligo and AA involve IFN-γ-mediated cytotoxicity via JAK1/JAK2 (1). This convergence on JAK-STAT signaling provides a rationale for a “multi-disease therapy” approach. The concurrent presence of severe AD, vitiligo, and AA in a single patient is exceptionally rare. Epidemiological data illustrate this rarity: the population prevalence of vitiligo is approximately 0.5% (2), and among these patients, only about 12.5% have comorbid AD (2). Furthermore, only about 3.2% of patients with AD have comorbid AA (3). The convergence of all three severe conditions therefore represents a low-probability clinical event, posing a significant therapeutic challenge, as traditional disease-specific therapies often lead to complex regimens, variable efficacy, and cumulative side effects (4). Upadacitinib, a selective JAK1 inhibitor, concurrently inhibits the downstream signaling of key cytokines (e.g., IL-4, IL-13, IFN-γ) implicated in all three diseases, offering a potential unified treatment strategy. However, systematic reports on its use in this specific triad are lacking.

This case report details a rare case of severe co-existing AD, vitiligo, and AA that improved rapidly with upadacitinib monotherapy. We describe the scientific basis for JAK-STAT targeting in “multi-disease therapy” and evaluate upadacitinib’s therapeutic potential in this challenging clinical scenario.

## Case presentation

### Clinical presentation and history

A 57-year-old man was admitted to our hospital in July 2024 with a chief complaint of generalized erythema and papules accompanied by severe pruritus for over one year. Prior treatments at local hospitals provided minimal relief. Physical examination revealed generalized confluent erythema with scaling. The estimated BSA involvement for atopic dermatitis was approximately 78%. Well-demarcated depigmented macules and patches were observed on the face, neck, trunk, and limbs, with an estimated cumulative BSA involvement for vitiligo of approximately 64%. Patchy hair loss was evident on the scalp and eyebrows. Palpable, non-tender lymph nodes were present in the bilateral inguinal and axillary regions.

### Diagnostic investigations

**Figure 1:**
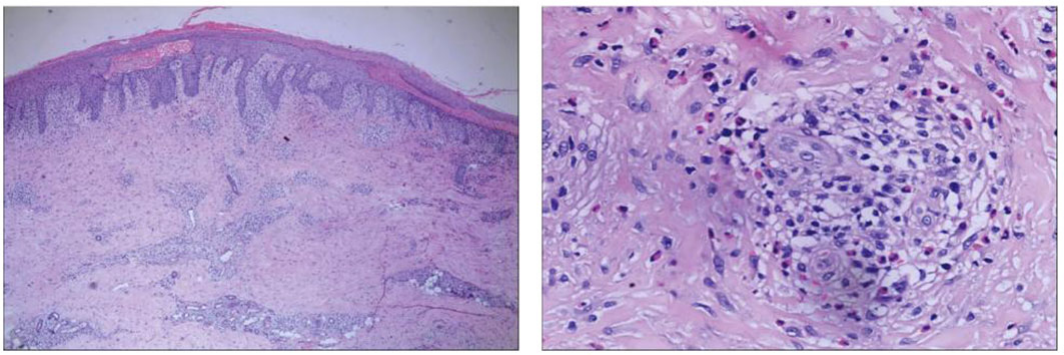
Histopathological findings. A skin biopsy from an erythematous lesion showed hyperkeratosis, spongiosis, serous exudation, and a perivascular inflammatory infiltrate of lymphocytes and eosinophils in the superficial dermis, consistent with AD.

Diagnostic assessments showed an elevated eosinophil count (1.07×10^9^/L), elevated total IgE (396.0 IU/mL), and elevated CRP (38.60 mg/L). Ultrasonography revealed reactive lymphadenopathy. A skin biopsy from an erythematous lesion showed epidermal changes included parakeratosis, serous exudation, acanthosis with spongiosis, and intraepidermal vesiculation, accompanied by irregular elongation of the rete ridges. Within the dermis, a perivascular inflammatory infiltrate, composed of a moderate number of lymphocytes and a scant number of eosinophils, was observed surrounding small blood vessels in the superficial to mid-dermis. (**Figure 1**). A left axillary lymph node biopsy confirmed dermatopathic lymphadenitis, ruling out cutaneous T-cell lymphoma. Other findings included benign hepatic nodules and renal cysts, which were deemed unrelated to the skin conditions. The final diagnoses were: 1. Severe Atopic Dermatitis; 2. Vitiligo; 3. Alopecia Areata; 4. Dermatopathic Lymphadenitis. These diagnoses were established based on comprehensive clinical evaluation and routine diagnostic procedures, including the histopathological examinations mentioned above. The disease severity at baseline was assessed retrospectively as: SCORing Atopic Dermatitis (SCORAD) index, 78.40; Vitiligo Area Scoring Index (VASI), 56.13; Severity of Alopecia Tool (SALT) score, 38.40%.

### Treatment course

The patient had an extensive history of prior treatments. This included systemic therapies such as corticosteroids administered orally or intravenously (methylprednisolone, dexamethasone), traditional Chinese herbal formulations with immunomodulatory properties (total glucosides of paeony, *Tripterygium wilfordii*), and compound glycyrrhizin, as well as various antihistamines (fexofenadine, ebastine, levocetirizine, ketotifen) and adjunctive medications (gabapentin, promethazine). Topical management comprised corticosteroids (e.g., fluticasone propionate cream) and calcineurin inhibitors (e.g., crisaborole ointment). Non-pharmacological interventions such as laser therapy, microwave irradiation, and ozone hydrotherapy were also attempted. This multi-modal regimen yielded unsatisfactory results. Consequently, on August 20, 2024, after screening and excluding contraindications, treatment with upadacitinib 15 mg once daily was initiated.

### Clinical outcomes

**Figure 2:**
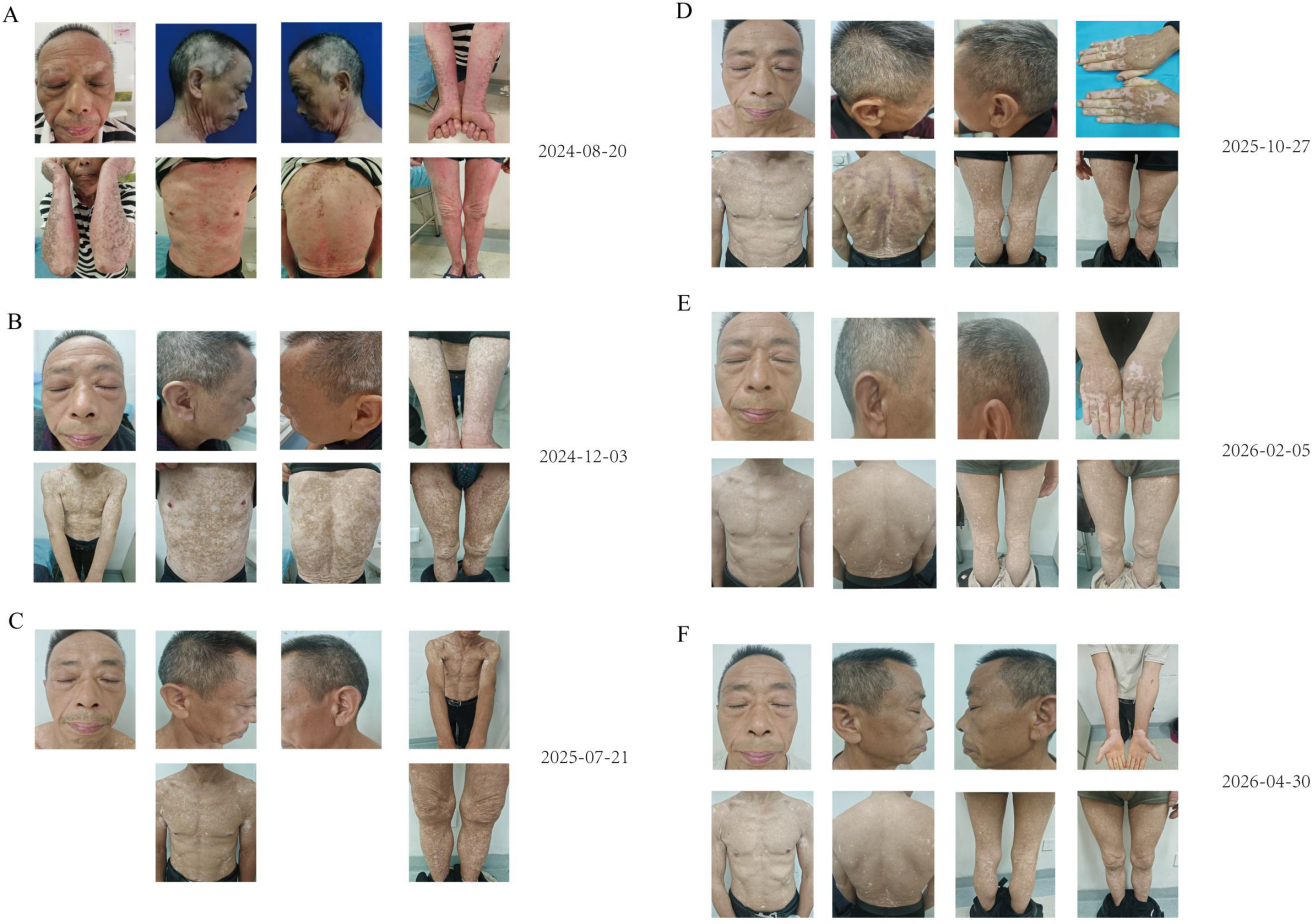
Longitudinal follow-up of skin manifestations and treatment response to upadacitinib.​ Serial clinical photographs from six time points illustrate the dynamic changes in atopic dermatitis (AD), vitiligo, alopecia areata (AA), and treatment-related hyperpigmentation. **(A)** Baseline (Aug 20, 2024): Severe, confluent erythema and scaling (AD) on the trunk and limbs, extensive well-demarcated depigmented patches of vitiligo on the face, neck, and trunk, and significant loss of eyebrow hair (AA). **(B)** 15 weeks (Dec 3, 2024): Marked clearance of AD lesions with residual post-inflammatory hyperpigmentation. Eyebrow hair begins to regrow, and pronounced hyperpigmentation is observed on the lower limbs and trunk. **(C)** 1 year (Jul 21, 2025): Sustained clearance of AD, substantial repigmentation of vitiligo lesions, and complete regrowth of eyebrow hair. **(D)** 1 year 2 months (Oct 17, 2025): New depigmented patches of vitiligo appear following a period of highly irregular dosing, while AD remains controlled. **(E)** 1.5 years (Feb 5, 2026) & **(F)** 1.6 years (Apr 30, 2026): With continued irregular dosing, AD control is maintained, vitiligo shows partial improvement, AA remission is sustained, and the hyperpigmentation exhibits further lightening.

**Figure 3:**
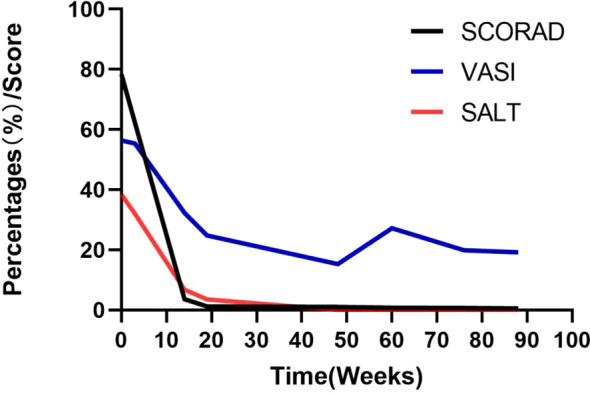
Longitudinal trends of disease severity scores during upadacitinib treatment. The graph depicts the changes in SCORing Atopic Dermatitis (SCORAD, black line), Vitiligo Area Scoring Index (VASI, blue line), and Severity of Alopecia Tool (SALT, red line) scores.

Treatment led to rapid and significant clinical improvement. For atopic dermatitis, pruritus subsided within 3–4 days and resolved completely after one week; erythema began to fade after 4–5 days and was nearly cleared by week 15, with the SCORAD score decreasing from a baseline of 78.40 to 3.62. Throughout the entire follow-up period until April 30, 2026, pruritus and erythema remained well-controlled even during phases of highly irregular medication adherence, with a SCORAD score of 0.52 at the final visit. For alopecia areata, eyebrow hair regrew completely within months, and the SALT score improved from a baseline of 38.40% to 0.00%, an effect sustained without relapse at the latest follow-up in April 2026. The sustained control of atopic dermatitis and alopecia areata stood in sharp contrast to the fluctuating activity of vitiligo under similar conditions of irregular dosing. Vitiligo activity proved to be highly sensitive to treatment adherence. During the period of regular upadacitinib (15 mg/day) administration, the patient’s condition improved markedly, with the VASI score decreasing from a baseline of 56.13 (August 20, 2024) to 15.32 after one year of treatment (July 21, 2025). However, after July 2025, when the patient’s actual drug intake became highly irregular, vitiligo recurred: at the follow-up on October 17, 2025, the VASI score increased to 27.18, and new depigmented patches were observed. Despite medical advice to resume regular dosing, the subsequent dosing frequency remained unstable (approximately twice a week). Later follow-ups on February 5 and April 30, 2026, yielded VASI scores of 19.87 and 19.23, respectively, indicating partial control of vitiligo activity that had not returned to the optimum level achieved during prior regular treatment (**Figure 2**). This suggests that maintaining remission in vitiligo may require more sustained and stable drug exposure compared to controlling atopic dermatitis. The longitudinal trends of these scores are consolidated in **Figure 3**, visually presenting the marked improvement and differential response patterns of all three diseases throughout the treatment course.

Notably, a distinct phenomenon of hyperpigmentation was observed during the treatment course. Approximately 10 days after the initiation of upadacitinib, hyperpigmentation began to develop on the patient’s lower legs and progressively extended upwards to the trunk and limbs. Pre-existing hyperpigmented areas also darkened. However, during subsequent follow-up (e.g., on July 21, 2025), when the patient reported irregular medication adherence (self-discontinuation or intermittent dosing), a spontaneous partial lightening of these hyperpigmented patches was observed (**Figure 2**).

### Safety monitoring and follow-up

Throughout the treatment, serial laboratory monitoring (including CBC, lipid profile, and LFTs) showed no abnormalities; no serious adverse events—specifically, no serious infections—occurred.

## Discussion

### Case significance and the “multi-disease therapy” paradigm

This case report presents a patient with a rare, severe co-occurrence of atopic dermatitis (AD), vitiligo, and alopecia areata (AA) who experienced rapid and significant improvement in all three conditions following monotherapy with the selective JAK1 inhibitor, upadacitinib. This successful therapeutic outcome provides clinical evidence supporting the efficacy of upadacitinib in this challenging context and prompts consideration of a re-evaluation of the management strategy for comorbid conditions sharing common immunopathological foundations—suggesting a potential shift from the traditional “disease-specific treatment” model towards a novel “multi-disease therapy” paradigm that targets shared mechanisms (1). The following discussion delves into the core mechanisms, clinical value, and future directions of this paradigm shift.

The concurrent presence of severe AD, vitiligo, and AA in a single patient presents a formidable challenge to conventional stepwise treatment protocols (5). A “divide-and-conquer” strategy often proves inadequate; for instance, cyclosporine might be used for AD, while other immunosuppressants or JAK inhibitors (e.g., tofacitinib) might be added for AA and vitiligo. Such polypharmacy not only risks antagonistic therapeutic effects but also leads to the accumulation of multiple side-effect risks, unpredictable drug interactions, and a significant increase in the patient’s medication burden, potentially resulting in an overall lower therapeutic index (4). The suboptimal response of our patient to initial conventional therapy exemplifies this dilemma. In contrast, upadacitinib, by precisely targeting the shared JAK-STAT signaling pathway hub, suggesting the potential for synergistic efficacy against multiple conditions, potentially circumventing the limitations of traditional approaches and thereby illustrating the rationale and potential of the “multi-disease therapy” concept.

### Scientific rationale and supporting evidence

Although AD, vitiligo, and AA manifest distinct clinical presentations, all involve aberrant activation of the JAK-STAT pathway, which may represent a convergent mechanism that could explain the concurrent therapeutic effect across distinct diseases. Although the downstream cytokine profiles differ (e.g., Th2 in AD vs. IFN-γ in vitiligo/AA), their signals converge on the JAK-STAT hub. In AD, the signaling of key cytokines central to its Th2-dominant inflammatory response—IL-4, IL-13, and the pruritogenic IL-31—heavily depends on JAK1 (6, 7). In AA, the process involving CD8+ T-cell attack on hair follicles implicates IFN-γ (signaling via JAK1/JAK2) and IL-15 (signaling via JAK1/JAK3) (8, 9). In vitiligo, the key driver IFN-γ, responsible for cytotoxic T-cell-mediated destruction of melanocytes, also relies on JAK1/JAK2 signaling (10, 11). Consequently, by highly selectively inhibiting JAK1, upadacitinib simultaneously downregulates these disparate cytokine signals at an upstream node. This provides a plausible mechanistic basis for the observed clinical synergy and supports the hypothesis that targeting a shared upstream pathway (JAK-STAT) can be effective across multiple diseases with distinct downstream mediators (12).

This strategy of “treating different diseases with the same method,” based on targeting a shared pathogenic hub, may have broad applicability. Similar “multi-disease therapy” models have been validated in other immune-mediated conditions, such as the disease cluster sharing the IL-23/Th17 axis (e.g., psoriasis, psoriatic arthritis, and Crohn’s disease) (13, 14). This established principle underscores the rationale for our approach.

The action of upadacitinib in this triad stems from its comprehensive suppression of pathogenic pathways via JAK-STAT inhibition, thereby reprogramming the local immune microenvironment.​ Its effect extends beyond merely suppressing inflammatory signals. In AD pathological models, the inhibition of JAK1 signaling has been shown to promote the expression of key epidermal differentiation proteins (e.g., filaggrin) and restore barrier function, a principle consistent with the observed clinical improvement in our patient (15). Crucially, it significantly reduces CXCR4-mediated T-cell migration and the infiltration of pathogenic CD8+ cytotoxic T cells into the skin, a common driver in all three conditions (16). In AA and vitiligo, by inhibiting IFN-γ signaling (dependent on JAK1/JAK2), upadacitinib reduces cytotoxic T-cell activity around hair follicles and melanocytes, aiding in the restoration of local immune tolerance (17). This broad suppression of pathogenic T-cell infiltration creates an inflammation-free milieu conducive to healing.

The long-term clinical efficacy of upadacitinib supports its potential for sustained disease control through such microenvironmental reprogramming. Data indicate that upadacitinib can maintain efficacy over the long term in diseases like AD and AA (18, 19). Furthermore, through sustained inhibition of IL-15 signaling (via JAK1/JAK3), it may clear or inhibit long-lived pathogenic T-cell populations in AA, thereby potentially reducing relapse risk (17). The resetting of immune homeostasis induced by long-term treatment may provide a “reset” opportunity for the immune system, potentially altering the long-term disease course (20).

### Pharmacological basis for differential treatment responses in coexisting AD, vitiligo, and AA

The differential therapeutic response observed in this case-sustained remission of atopic dermatitis (AD) versus recurrence of vitiligo during dose reduction or irregular dosing-offers a valuable clinical perspective on the pharmacological variances of JAK inhibitors across different immune-mediated dermatoses. These differences are primarily manifested in the kinetics of onset, required treatment intensity, and threshold for maintaining remission.

In the treatment of AD, JAK inhibitors are characterized by their rapid onset of action. Oral highly selective JAK1 inhibitors (e.g., upadacitinib) can significantly alleviate pruritus within days and lead to objective improvement of skin lesions within 2–4 weeks (21, 22), attributable to their swift blockade of key AD cytokine signals (IL-4, IL-13, IL-31) (23). Consequently, 15 mg once daily has been established as the standard effective dose for moderate-to-severe AD (21, 24).

In contrast, the treatment of vitiligo and alopecia areata (AA) typically requires a longer time to onset and more sustained, stable drug exposure. Achieving clinically significant repigmentation in vitiligo often necessitates continuous treatment for over 6 months (25), as it involves not only suppressing IFN-γ-mediated immune attack (26), but also awaiting the slow process of melanocyte functional recovery. Although effective in AA treatment, substantial hair regrowth often takes months, and due to a high risk of relapse, long-term or even continuous dosing is frequently required to maintain efficacy (27, 28). This stems from their pathogenic mechanisms involving long-lived immune memory and destruction of specific target cells (melanocytes, hair follicles) by cytotoxic T cells, halting this process and restoring target cell function requires deeper and more durable immune modulation (26, 29).

Thus, the disease course in this case provides direct validation of the aforementioned pharmacological differences. The standard daily dose effective for AD remained sufficient to control its acute inflammation during “de facto dose reduction” due to poor adherence, yet failed to meet the higher threshold required to maintain remission in vitiligo, leading to its recurrence. This finding carries a central implication for the “multi-disease therapy” paradigm: when using a single pathway inhibitor to treat multiple comorbidities, the dosing strategy must be prioritized to satisfy the condition with the highest “maintenance treatment threshold” (vitiligo in this case), rather than targeting only the “fastest-responding” one (AD). This necessitates that clinical decisions regarding initial and maintenance regimens be guided by the most recalcitrant comorbidity, with particular emphasis on patient education to ensure long-term, regular treatment adherence, thereby optimizing the overall therapeutic outcome.

### Treatment-associated hyperpigmentation: phenomenon and hypothesis

The dynamic hyperpigmentation observed in this patient following irregular dosing may represent post-inflammatory hyperpigmentation after AD resolution, where cytokines like TNF-α stimulate melanocytes (30). As the drug’s primary target, JAK-STAT pathway inhibition may also indirectly affect pigmentation by altering the immune microenvironment (31, 32). A key intersecting mechanism is the SCF/c-Kit axis, vital for melanocyte function and known to interact with JAK-STAT signaling; its modulation by upadacitinib could influence melanocyte activity (33). Although hyperpigmentation is a rare side effect of other kinase inhibitors (e.g., imatinib) (34), such pronounced, dose-dependent changes are not commonly reported in upadacitinib trials for AD. This novel presentation, likely arising from the unique context of severe AD with vitiligo, underscores the need to clarify the role of JAK-STAT signaling in pigmentation regulation, particularly in comorbid disease states.

### Safety considerations

In this case, upadacitinib was well-tolerated, with no serious adverse events (e.g., infections, herpes zoster) reported during the follow-up period. Serial monitoring of laboratory safety parameters, including complete blood count, lipid profile, and liver function tests, revealed no clinically significant abnormalities. This favorable safety observation aligns with the established tolerability profile of upadacitinib in clinical trials for atopic dermatitis (34). Nevertheless, it is important to contextualize this individual outcome within the known class-wide risks associated with JAK inhibitors. Large-scale studies have identified an increased risk of serious infections, herpes zoster, and for some agents, potential elevations in major adverse cardiovascular events (MACE) and malignancy compared to TNF inhibitors, particularly in high-risk populations (35). The absence of such signals in our patient may be attributed to the relatively short follow-up duration, the absence of traditional cardiovascular risk factors, and the specific clinical context of this single case. Therefore, this favorable outcome should be interpreted cautiously. The benefit-risk assessment for JAK inhibitors necessitates individualized evaluation of patient factors (35). While this case contributes real-world evidence supporting the tolerability of upadacitinib in a complex comorbid scenario, it simultaneously reinforces the imperative for ongoing, guideline-recommended monitoring in clinical practice.

### Consideration of spontaneous remission and confounding factors

The natural variability of AD, vitiligo, and AA necessitates considering alternative explanations for the observed improvement. However, spontaneous remission is unlikely as the primary cause. The rapid, simultaneous, and marked regression of all three severe diseases immediately followed upadacitinib initiation, a highly improbable coincidence. This is further supported by objective, dramatic reductions in SCORAD, VASI, and SALT scores. Prior ineffective therapies were discontinued at baseline, and no other new interventions were introduced, minimizing confounding. The dose-dependent dynamic hyperpigmentation also indicates a direct drug effect. While unmeasured factors cannot be excluded, the evidence collectively supports the central therapeutic role of JAK1 inhibition.

### Clinical implications

This case supports the rationale for considering a shift in clinical practice from the traditional “disease-specific treatment” to a “multi-disease therapy” paradigm. For patients with complex comorbidities like this one, traditional multi-drug combination regimens lead to challenges including inconsistent efficacy, cumulative side effects, and management complexity. The mono-therapy strategy with upadacitinib achieves maximized treatment efficiency, minimized risk of side effects, and optimized patient quality of life. If validated, this approach necessitates a new framework for benefit-risk assessment: it must be based on the combined efficacy across all comorbidities. This is because the holistic benefit​ of controlling multiple severe diseases with one agent may outweigh its risks, leading to a favorable overall therapeutic index compared to managing each disease separately. Consequently, clinical decisions should prioritize the patient’s total net benefit.

### Limitations, and future directions

As a single case report, this study has inherent limitations, including the lack of a control group and a relatively short observation period. Larger prospective studies and longer follow-up times are necessary to validate the general applicability, long-term safety, and durability of this strategy after treatment cessation. Furthermore, serial measurements of specific inflammatory biomarkers such as IgE and CRP were not available during follow-up, which could have provided additional objective data on the treatment. Future research directions should include: (1) designing targeted clinical trials with enrollment criteria specifically defining patients having at least two of the following: AD, vitiligo, or AA, to verify the general applicability of this strategy; (2) investigating pre-treatment cytokine profiles (e.g., levels of IFN-γ, IL-4, IL-13), proportions of specific immune cell subsets, or JAK-STAT pathway-related gene polymorphisms in patient blood or skin tissue (36), to achieve precise patient stratification; (3) optimizing treatment strategies (e.g., combination therapies, dosing intervals) to balance long-term efficacy with safety.

## Conclusion

This case suggests that upadacitinib monotherapy could represent a promising strategy of ‘treating different diseases with the same method’ for patients with severe co-existing AD, vitiligo, and AA driven by a shared JAK-STAT pathway. It provides preliminary clinical support for the ‘multi-disease therapy’ paradigm, which targets common pathogenic mechanisms, and points to a new direction for the management of refractory immune-mediated comorbid conditions that warrants further investigation.

## Data Availability

The original contributions presented in the study are included in the article/supplementary material. Further inquiries can be directed to the corresponding author.
